# Dietary supplementation with *Bacillus subtilis* KC1 alleviates the negative effects of *Mycoplasma gallisepticum* on growth performance and amino acid metabolism of broiler chickens

**DOI:** 10.3389/fvets.2024.1477575

**Published:** 2024-10-23

**Authors:** Xueping Chen, Jiayao Cui, Yuanyuan Wang, Keguang Han, Nairui Huo, Jian Wang

**Affiliations:** College of Veterinary Medicine, Shanxi Agricultural University, Taigu, China

**Keywords:** *Bacillus subtilis*, *Mycoplasma gallisepticum*, amino acid, metabolomics, transcriptomics

## Abstract

The aim of this study was to explore whether and how *Bacillus subtilis* KC1 can enhance the growth performance of *Mycoplasma gallisepticum* (MG)-infected broilers. Broilers were randomly divided into 4 groups: the control group (basal diet), the MG group (basal diet + MG challenge), the KC group (basal diet + *B. subtilis* KC1 supplementation), the KC + MG group (basal diet + *B. subtilis* KC1 supplementation + MG challenge). The results showed that, compared to the control group, MG group exhibited significantly reduced body weight and average daily gain, and increased feed conversion ratio of broilers. However, compared to the MG group, the *B. subtilis* KC1 + MG group exhibited significantly improved above indicators of growth performance. In addition, compared to the MG group, *B. subtilis* KC1 + MG group exhibited increased superoxide dismutase levels and reduced levels of malondialdehyde, interleukin-1β, and tumor necrosis factor-*α* of broilers. Furthermore, metabolomics and transcriptomics analyses indicated that MG infection disrupted amino acid metabolism in broilers, whereas *B. subtilis* KC1 supplementation alleviated the abnormal amino acid metabolism caused by MG. These results suggested that *B. subtilis* KC1 may alleviate the poor growth performance caused by MG infection in broilers by improving amino acid metabolism.

## 1 Introduction

Among the various avian pathogens, *Mycoplasma gallisepticum* (MG) stands out as a major reason for the occurrence of chronic respiratory disease in chickens (1). A particularly worrying aspect is the capacity of MG infections to spread both vertically and horizontally (1, 2). Chickens infected with MG experience a range of performance and production losses, including decreased egg production, reduced hatchability rates, compromised feed conversion efficiency, and diminished weight gain, ultimately resulting in significant economic losses for poultry industries worldwide (1, 2). Furthermore, chickens infected with MG become immunosuppressed, and thus more susceptible to other pathogens, ultimately exacerbating the economic losses suffered by farmers (3).

As a type of feed additive, probiotics offer numerous benefits, including improving growth performance, maintaining intestinal health, and enhancing the immunity of chickens (4–6). However, the intolerance of most probiotics to gastric acid and high temperature poses challenges for their application in poultry production. *Bacillus subtilis* is capable of spore formation in harsh environments and possesses unique biological properties, including resistance to gastric acid and high temperature (7, 8). Additionally, being an aerobic bacterium, it consumes a considerable amount of free oxygen during reproduction in the intestinal tract, thereby significantly promoting the growth of anaerobic probiotics like *Lactobacillus* and *Bifidobacterium* (9, 10). *Bacillus subtilis* is often used as a feed additive because of its properties of improving growth performance and enhancing immunity (11–13). For example, compared to the control group, dietary supplementation with *B. subtilis* BYS2 significantly increased average weight and immune organ indices of broilers (11). Furthermore, dietary supplementation with *B. subtilis* BYS2 significantly reduced the mortality rate of *Escherichia coli-* and Newcastle disease virus-infected chickens (11). Therefore, it is necessary to screen *B. subtilis strains* that have the potential to enhance the resistance of chickens to MG.

Metabolomics is a research approach that quantitatively analyzes all metabolites in a biological organism (14). Its research objects are mostly small molecules with relatively low molecular weights, such as organic acids, amino acids, sugars, lipids and vitamins (15). Metabolomics can assist researchers in gaining a more comprehensive understanding of the metabolic characteristics of biological systems and elucidating the physiological or pathological mechanisms that underlie phenotypic changes. The narrow definition of the transcriptome refers to the collection of all mRNA molecules that are produced by the expression of all genes within a biological organism under a specific physiological condition. Changes in the transcriptome can influence changes in the metabolome. Integrative analysis of the transcriptome and metabolome can help provide a more comprehensive understanding of the mechanisms underlying a specific phenotype in a biological organism. A strain of *B. subtilis* KC1 was previously isolated from healthy chicken feces, and the *B. subtilis* KC1 exhibited good probiotic potential, including acid and alkali resistance, as well as the ability to inhibit the growth of serval intestinal pathogens (16). In this study, the protective effects of *B. subtilis* KC1 on the growth performance of chickens infected with MG were evaluated. Furthermore, the underlying mechanism of *B. subtilis* KC1 against MG-infected chickens was investigated using metabonomics and transcriptomics. The present study demonstrated that *B. subtilis* KC1 alleviates the poor growth performance induced by MG infection, and the underlying mechanism involves the improvement of amino acid metabolism.

## 2 Materials and methods

### 2.1 Animals

Broilers (male, 1-day-old) of the Arbor Acres (AA) breed were acquired from a nearby poultry farm and reared in hygienic housing with consistent light/dark cycles, provided with water and feed *ad libitum*. All the broilers underwent testing, and none of them were found to have MG. When the trial was over, all chickens were euthanized by cervical dislocation.

### 2.2 Experimental design

A total of 96 one-day-old male AA broilers with an initial average body weight (39.29 ± 0.54 g) were selected and divided into 4 groups: (1) broilers were fed a control diet (Con); (2) broilers were fed a control diet supplemented with 10^8^ CFU/kg of *B. subtilis* KC1 (KC); (3) broilers were fed a control diet and challenged with MG at day 14 (MG); and (4) broilers were fed a control diet supplemented with 10^8^ CFU/kg of *B. subtilis* KC1, and further challenged with MG at day 14 (MG_KC). Each treatment was replicated 3 times, with 8 broilers per replicate. All diets were formulated to meet the National Research Council and Chinese chicken feeding standards (NY/T-33–2004) (17, 18). A single-layer, three-dimensional chicken coop was used. Temperature was gradually reduced by 2°C per week from 33 to 23°C and then kept constant.

### 2.3 MG culture and MG infection procedure

The MG strain *R*_low_ was obtained from the China Veterinary Culture Collection Center and cultured in a *Mycoplasma* medium (H910KJ, BasalMedia, Shanghai, China), supplemented with 1% ampicillin (ST008, Beyotime, Shanghai, China) and 10% fetal bovine serum (S660JJ, BasalMedia, Shanghai, China). The procedure for infecting the broilers with MG involved the use of a syringe to inoculate a 200 μL volume of solution, containing 2 × 10^9^ color change units (CCUs) of MG, into the left air sacs of the broilers.

### 2.4 *Bacillus subtilis* preparation

The strain of *B. subtilis* KC1 (GenBank accession number OL721931) was previously isolated from the fecal matter of healthy chickens (16). This *B. subtilis* KC1 strain was cultured in a modified liquid medium, composed of 1.25 g of glucose, 1.25 g of yeast extract, 1.25 g of peptone, 1.25 g of beef paste, and 1.25 g of NaCl dissolved in 250 mL of sterilized water, adjusted to a pH of 7.2, to achieve a bacterial suspension of 10^8^ CFU/mL. Subsequently, the *B. subtilis* KC1 was mixed fully with the control diet, achieving a final concentration of 10^8^ CFU per kilogram of feed.

### 2.5 Growth performance

The broilers of each replicate were weighed at the beginning and day 42. Feed intake and the number of broilers were recorded to calculate the average daily gain (ADG), average daily feed intake (ADFI) and feed conversion ratio (FCR) of every replicate. ADG = [total body gain (g)/test days]/number of broilers in each replicate, ADFI = [total feed intake (g)/test days]/number of broilers in each replicate, FCR = ADFI/ADG.

### 2.6 Serum parameters

Blood samples were collected (6 samples per treatment, 2 per replicate) at day 42. Serum was obtained by centrifuging the blood at 3,000 × *g* for 10 min at 4°C. Following the manufacturer’s instructions, serum superoxide dismutase (SOD, A001-3-2, Nanjing Jiancheng Biotechnology Co., Ltd., Nanjing, China), malondialdehyde (MDA, A003-1-2, Nanjing Jiancheng Biotechnology Co., Ltd., Nanjing, China), interleukin-1 (IL-1β, ml002787, Shanghai Enzyme-linked Biotechnology Co., Ltd., Shanghai, China) and tumor necrosis factor-a (TNF-*α*, ml002790, Shanghai Enzyme-linked Biotechnology Co., Ltd., Shanghai, China) levels were detected using commercially ELISA kits.

### 2.7 Untargeted metabolomics

Serum was obtained as described above. Untargeted metabolomics was completed by Beijing Genomics Institute (BGI, Shenzhen, China). To prepare the samples for metabolomic analysis, add 100 μL of serum from each sample to a tube. Subsequently, add 700 μL of an extractant containing internal standard (prepared at a ratio of methanol: acetonitrile: water = 4:2:1, V/V/V), shake vigorously for 60 s, and then place the tubes in a −20°C refrigerator for 2 h. After the incubation, centrifuge the samples at 25,000 × *g* for 15 min at 4°C. Carefully remove the samples from the centrifuge and transfer 600 μL of the supernatant into a new tube. Proceed with drying using a drying machine until complete. Once dry, add 180 μL of a methanol: pure water mixture (1,1, v/v), vortex for 10 min to ensure complete dissolution in the reconstituted solution. Centrifuge the samples again at 25,000 × *g* for 15 min at 4°C. Collect the supernatant into a fresh tube. Take 20 μL of each sample and combine them to create quality control (QC) samples. Finally, transfer the prepared supernatants to the LC–MS/MS analysis steps. The sample extracts were analyzed using Waters UPLC I-Class Plus (Waters, United States) equipped with QTRAP 6500 Plus (SCIEX, United States). After importing the off-line data of mass spectrometry into compound discoverer 3.3 (Thermo Fisher Scientific, United States) software and analyzing the mass spectrometry data in combination with BMDB (BGI metabolome database), mzcloud database and chemspider online database, a data matrix containing information such as metabolite peak area and identification results will be obtained. These metabolites were annotated using the KEGG database and HMDB database. The principal component analysis (PCA) and orthogonal partial least squares-discriminant analysis (OPLS-DA) were performed at meta X. Metabolites were considered to be significantly different if they had a variable important for the projection (VIP) value greater than 1, a *p-*value less than 0.05, and a fold change of at least 1.2 or not more than 0.83. Volcano plots were then generated based on the log2 of the fold change and the negative log10 of the *p-*value for these metabolites. The functions of these differential metabolites and their associated metabolic pathways were investigated using the KEGG database.

### 2.8 Transcriptomics

Lung, liver and ileum samples were collected (3 samples per treatment, 1 per replicate) at day 42. Total RNA was extracted using TRIzol reagent (ET101-01-V2, Beijing TransGen Biotech Co., Ltd., Beijing, China). The RNA concentration and integrity were detected using a Fragment Analyzer (Agilent, California, United States). RNA samples that complied with the criteria of having a concentration of more than 40 ng/μL, an integrity value greater than 7.0, and a total content of 1 μg or more were chosen for further experimentation. Transcriptomic sequencing was completed by BGI. The procedures for library construction, sequencing and bioinformatics analysis were as follows. Oligo dT beads were employed to enrich mRNA carrying a poly(A) tail. Subsequently, the RNA was fragmented, and first-strand cDNA production was initiated through random N6-primed reverse transcription. This was followed by the synthesis of second-strand cDNA, where dUTP was utilized instead of dTTP. The resulting cDNA then underwent end-repair and subsequent 3′ adenylation. Afterward, adaptors were attached to the termini of these 3′ adenylated cDNA segments. Before initiating PCR amplification, the strand marked with dUTP was selectively degraded using Uracil-DNA-Glycosylase (UDG). The remaining strand was then amplified to create a cDNA library suitable for sequencing. To enrich the purified cDNA template, multiple rounds of PCR amplification were carried out using PCR primers. Heat was used to denature the PCR product, and subsequently, the single-stranded DNA was circularized with the aid of a splint oligo and DNA ligase. Ultimately, DNA nanoball synthesis and sequencing were executed on the DNBSEQ platform in paired-end 150 sequencing mode. Sequencing data were called raw reads, and quality control was then performed on the raw reads to obtain clean reads. After quality control, the filtered clean reads were aligned to the reference sequence. Then, gene expression quantification, gene difference analysis, and enrichment analysis were performed. Data quality control was completed using SOAPnuke (v1.5.2). Genome alignment was completed using HISAT2 (2.0.4). Transcript quantification and differential comparison were completed via RSEM and DESeq2, respectively. Genes were considered to be significantly different if they had a *q-*value (adjusted *p-*value) less than 0.05 (19). Volcano plots were then generated based on the log2 of the fold change and the negative log10 of the *q-*value. The functions of these differential genes and their associated metabolic pathways were investigated using the KEGG database.^1^

### 2.9 Statistical analysis

The results were presented as mean ± standard deviation (SD) and were analyzed utilizing GraphPad Prism 8.0 software (GraphPad Software, Inc.). The comparisons among multiple groups were conducted using one-way analysis of variance (ANOVA) accompanied by Tukey’s multiple comparison test. A *p-*value less than 0.05 was deemed to indicate statistical significance.

## 3 Results

### 3.1 *Bacillus subtilis* KC1 enhanced the growth performance of MG-infected chickens

**Figure 1:**
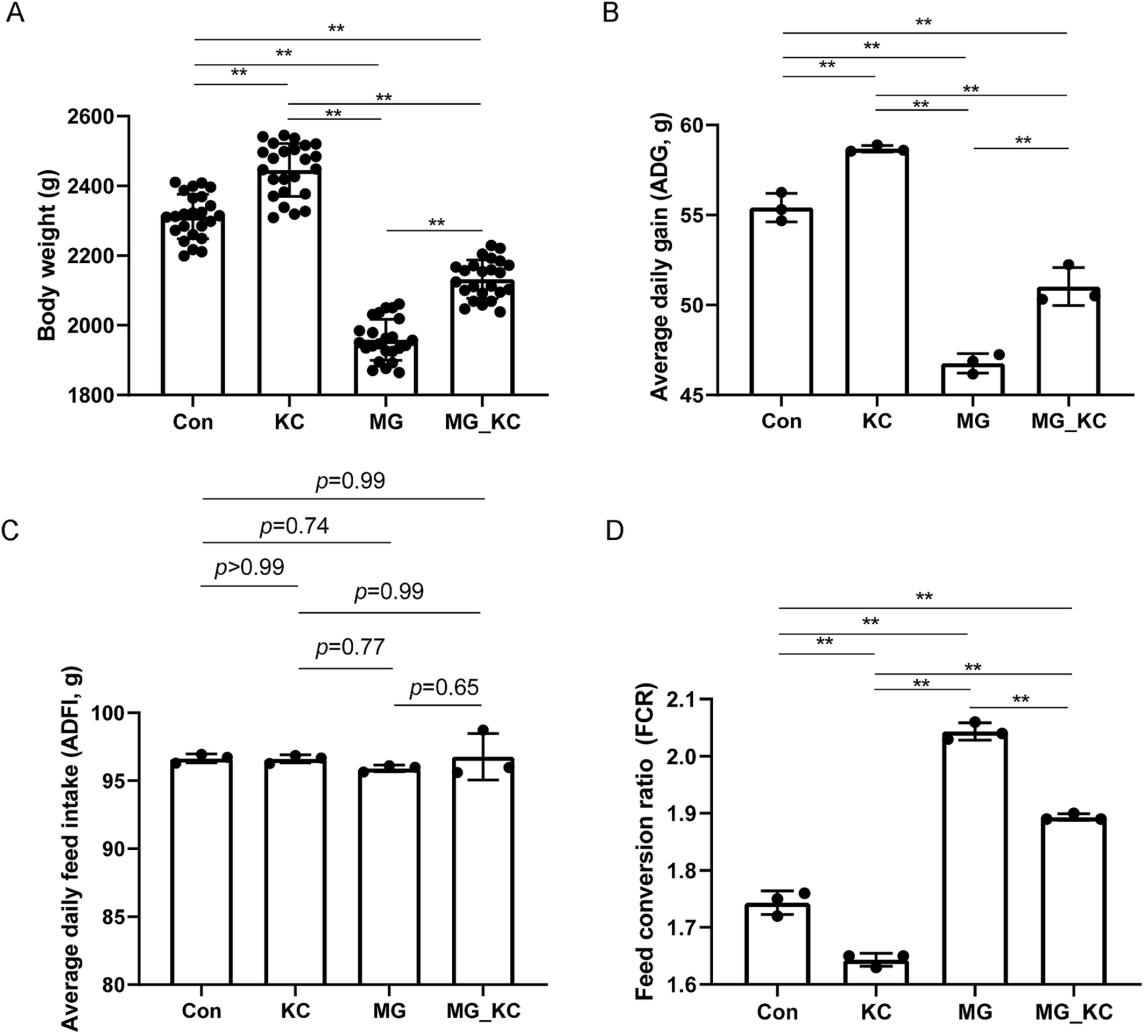
Effect of dietary *Bacillus subtilis* KC1 supplementation on growth performance of MG-infected broilers. **(A)** Body weight of broilers (*n* = 24). **(B)** Average daily gain (ADG, *n* = 3). **(C)** Average daily feed intake (ADFI, *n* = 3). **(D)** Feed conversion ratio (FCR; *n* = 3). **p < 0.01 indicates statistical significance. Con, control group; KC, *Bacillus subtilis* KC1 group; MG, *Mycoplasma gallisepticum* group; MG_KC, *Mycoplasma gallisepticum + Bacillus subtilis* KC1 group.

Compared with the Con group, the KC group exhibited significantly increased final body weight (*p* < 0.0001) and ADG (*p* = 0.0023) of broilers, as well as significantly decreased FCR (*p* = 0.0019) (Figures 1A,B,D). Compared with the Con group, the MG group exhibited significantly reduced final body weight (*p* < 0.0001) and ADG (*p* < 0.0001) of broilers, and significantly increased FCR (*p* < 0.0001) (Figures 1A,B,D). Compared with the MG group, the MG_KC group exhibited significantly increased final body weight (*p* < 0.0001) and ADG (*p* = 0.0034) of broilers, and significantly reduced FCR (*p* < 0.0001) (Figures 1A,B,D). There was no significant difference in ADFI among all groups (Figure 1C). These results indicated that dietary *B. subtilis* KC1 supplementation improved the growth performance of MG-infected chickens.

### 3.2 *Bacillus subtilis* KC1 improved the serum parameters associated with oxidative stress and inflammatory response in MG-infected broilers

**Figure 2:**
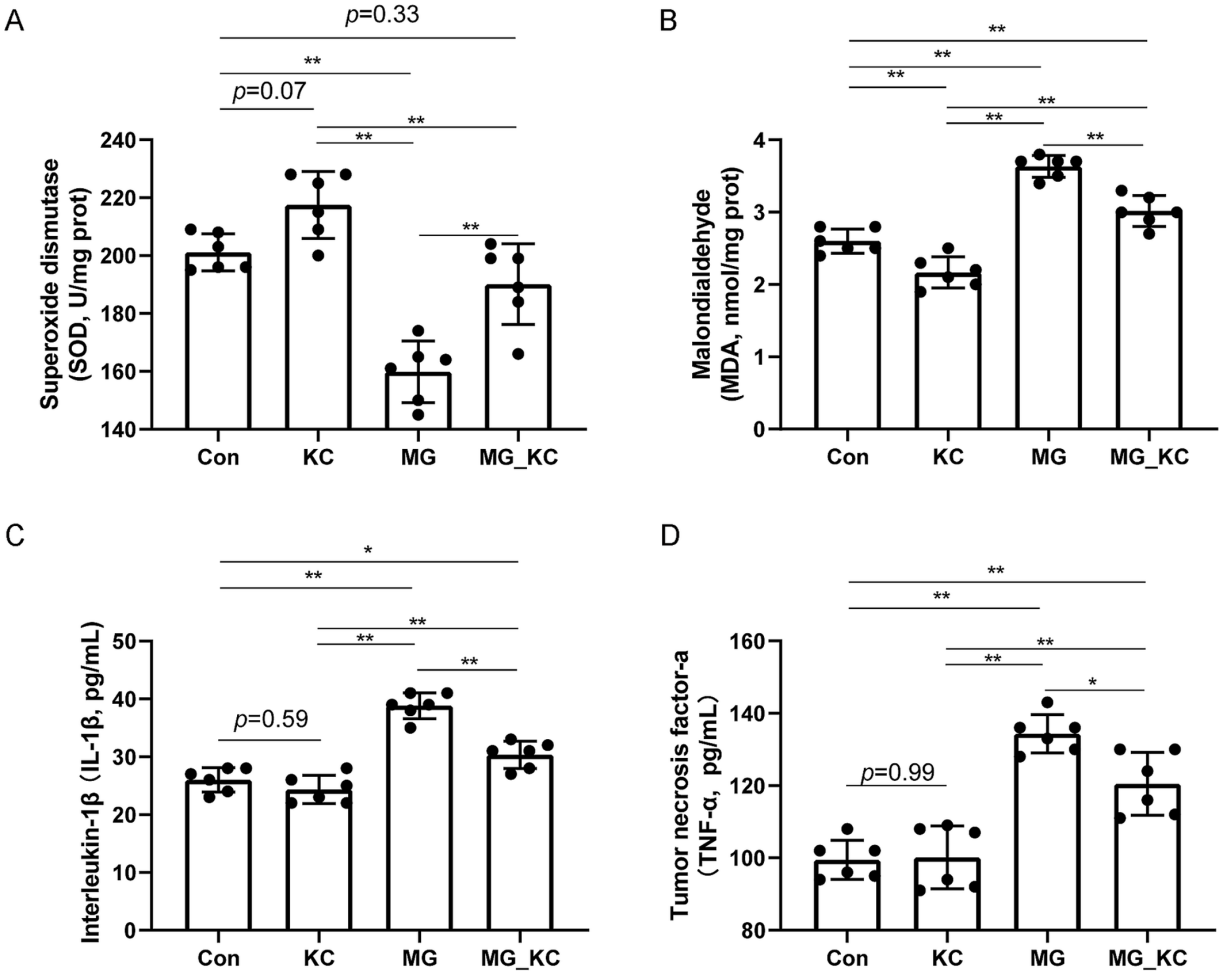
Effect of dietary *Bacillus subtilis* KC1 supplementation on markers of oxidative stress and inflammatory response of MG-infected broilers. **(A)** Superoxide dismutase (SOD) content (*n* = 6). **(B)** Malondialdehyde (MDA) content (*n* = 6). **(C)** Interleukin-1β (IL-1β) content (*n* = 6). **(D)** Tumor necrosis factor-a (TNF-*α*) content (*n* = 6). *p < 0.05, **p < 0.01 indicate statistical significance. Con, control group; KC, *Bacillus subtilis* KC1 group; MG, *Mycoplasma gallisepticum* group; MG_KC, *Mycoplasma gallisepticum + Bacillus subtilis* KC1 group.

Compared with the Con group, the KC group exhibited a increased trend in SOD activity (*p* = 0.0770) and significantly reduced MDA content (*p* = 0.0030) in serum (Figures 2A,B). Compared with the Con group, the MG group exhibited significantly reduced SOD activity (*p* < 0.0001) and significantly increased MDA content (*p* < 0.0001) in serum (Figures 2A,B). Compared with the MG group, the MG_KC group exhibited significantly increased SOD activity (*p* = 0.0006) and significantly reduced MDA content (*p* < 0.0001) in serum (Figures 2A,B).

Compared with the Con group, the KC group did not affect the levels of IL-1β (*p* = 0.5922) and TNF-*α* (*p* = 0.9985) (Figures 2C,D). Compared with the Con group, the MG group exhibited significantly increased serum IL-1β (*p* < 0.0001) and TNF-α levels (*p* < 0.0001) (Figures 2C,D). Compared with the MG group, the MG_KC group exhibited significantly reduced serum IL-1β (*p* < 0.0001) and TNF-α levels (*p* = 0.0164) (Figures 2C,D). These results indicated that dietary *B. subtilis* KC1 supplementation alleviated the oxidative stress and pro-inflammatory response caused by MG.

### 3.3 Metabolomics revealed *Bacillus subtilis* KC1 alleviated abnormal amino acid metabolism of MG-infected chickens

**Figure 3:**
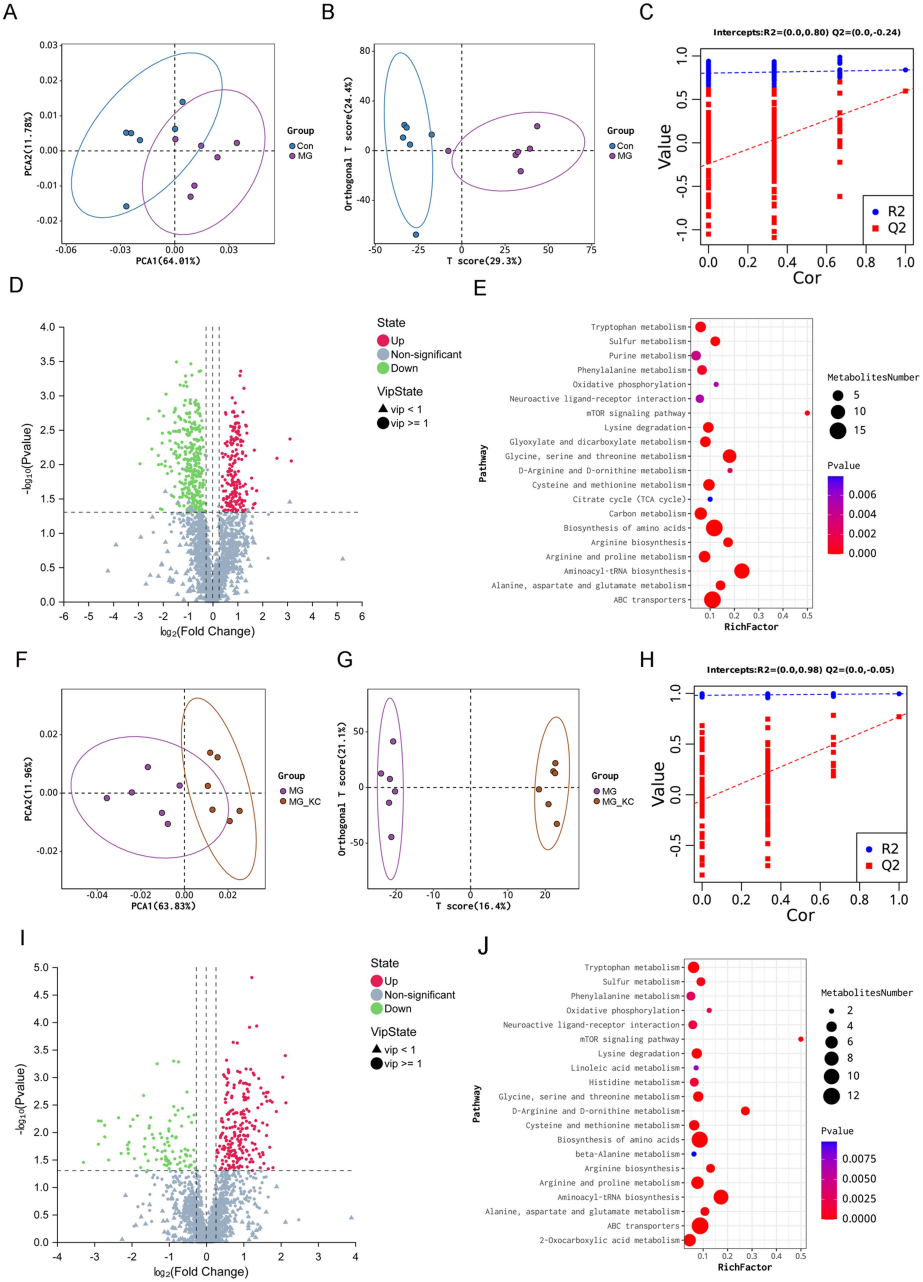
Effect of dietary *Bacillus subtilis* KC1 supplementation on the metabolic profiles of the serum samples from MG-infected broilers. **(A)** Principle component analysis (PCA) was performed on Con and MG groups (*n* = 6). **(B)** Orthogonal partial least squares-discriminant analysis (OPLS-DA) was performed on Con and MG groups (*n* = 6). **(C)** Cross-validation plot with a permutation test repeated 200 times. The intercepts of R2 = (0.0, 0.80) and Q2 = (0.0, −0.24), indicate that the OPLS-DA model is not overfitting (*n* = 6). **(D)** Volcano plots showed the results of the pairwise comparisons of serum metabolites in the Con and MG groups (*n* = 6). The significant metabolites are presented in red (up-regulated) or green (down-regulated). **(E)** The pathway enrichment analysis of significantly different metabolites in the Con and MG groups (*n* = 6). **(F)** PCA was performed on MG and MG_KC groups. **(G)** OPLS-DA was performed on MG and MG_KC groups (*n* = 6). **(H)** Cross-validation plot with a permutation test repeated 200 times. The intercepts of R2 = (0.0, 0.98) and Q2 = (0.0, −0.05), indicate that the OPLS-DA model is not overfitting (*n* = 6). **(I)** Volcano plots showed the results of the pairwise comparisons of serum metabolites in the MG and MG_KC groups (*n* = 6). The significant metabolites are presented in red (up-regulated) or green (down-regulated). **(J)** The pathway enrichment analysis of significantly different metabolites in the MG and MG_KC groups (*n* = 6). Con, control group; KC, *Bacillus subtilis* KC1 group; MG, *Mycoplasma gallisepticum* group; MG_KC, *Mycoplasma gallisepticum + Bacillus subtilis* KC1 group.

A total of 4,579 annotated metabolites were identified (Supplementary File S1). The PCA indicated that the serum samples from the MG group were significantly separated from the Con group (Figure 3A). Moreover, distinct clusters from the MG group compared with the Con group were confirmed by the OPLS-DA (Figure 3B). To assess the quality of the model without the risk of overfitting, 200 response permutation tests (RPT) were performed on the OPLS-DA model. The results indicated that the model was reliable and did not suffer from overfitting, as evidenced by the fact that the intercept of the goodness-of-fit measure (R2) was greater than the intercept of the goodness-of-prediction measure (Q2), and furthermore, the intercept of Q2 was less than zero (Figure 3C). Total 531 significantly differential metabolites were identified in the MG group compared with the Con group (Supplementary File S2). The volcano plots illustrated the global distribution of metabolites, revealing distinct differences in metabolite levels between the Con and MG groups (Figure 3D). KEGG pathway enrichment analysis indicated that the differential metabolites mainly enriched in the amino acids metabolism related pathways (Figure 3E).

The PCA indicated that the serum samples from the MG_KC group were significantly separated from the MG group (Figure 3F). Moreover, distinct clusters from the MG_KC group compared with the MG group were confirmed by the OPLS-DA (Figure 3G). In order to judge the quality of the model without fitting risk, 200 response permutation tests (RPT) were performed on the OPLS-DA model. The results indicated that the model was reliable and did not suffer from overfitting, as evidenced by the fact that the R2 was greater than the Q2, and furthermore, the intercept of Q2 was less than zero (Figure 3H). Total 318 significantly differential metabolites were identified in the MG_KC group compared with the MG group (Supplementary File S3). The volcano plots illustrated the global distribution of metabolites, revealing distinct differences in metabolite levels between the MG and MG_KC groups (Figure 3I). KEGG pathway enrichment analysis indicated that the differential metabolites mainly enriched in the amino acids metabolism related pathways (Figure 3J).

Compared with the Con group, the MG group exhibited significantly reduced l-proline, l-threonine, l-histidine, l-lysine, l-serine, l-aspartic acid, l-arginine, l-glutamine, Leucine, l-tryptophan, glycine, asparagine, *O*-acetyl-l-serine, l-Homoserine and l-allo-threonine levels (Table 1). Compared with the MG group, the MG_KC group exhibited significantly increased l-proline, l-histidine, l-lysine, l-aspartic acid, l-arginine, leucine, l-tryptophan, asparagine and l-homoserine (Table 1). Above all, these results indicated that MG infection induced abnormal amino acid metabolism, while *B. subtilis* KC1 alleviated the abnormal amino acid metabolism caused by MG.

**Table 1:** Effect of dietary *Bacillus subtilis* KC1 supplementation on the serum amino acids levels of MG-infected broilers.

Name	Fold change (MG/Con)	Fold change (MG_KC/MG)	*P*-value (MG/Con)	*P-*value (MG_KC/MG)	VIP (MG/Con)	VIP (MG_KC/MG)
l-proline	0.39	1.68	0.002	0.027	1.575	1.553
l-threonine	0.53	1.44	0.013	0.101	1.348	1.093
l-histidine	0.35	1.91	0.002	0.014	1.524	1.670
l-lysine	0.53	1.82	0.009	0.009	1.364	1.920
l-serine	0.52	1.30	0.020	0.248	1.261	0.784
l-aspartic acid	0.64	1.75	0.010	0.029	1.498	1.432
l-arginine	0.60	1.71	0.009	0.007	1.286	1.885
l-glutamine	0.55	1.20	0.018	0.270	1.322	0.749
Leucine	0.62	1.52	0.001	0.001	1.612	2.122
l-tryptophan	0.63	1.67	0.003	0.002	1.464	1.867
Glycine	0.71	1.10	0.034	0.407	1.168	0.628
Asparagine	0.40	1.80	0.018	0.043	1.245	1.354
*O*-acetyl-l-serine	0.64	1.06	0.025	0.577	1.201	0.458
l-homoserine	0.43	1.83	0.006	0.045	1.500	1.407
l-allo-threonine	0.52	1.45	0.019	0.193	1.316	1.051

### 3.4 Transcriptomics revealed *Bacillus subtilis* KC1 alleviated abnormal amino acid metabolism of MG-infected chickens

**Figure 4:**
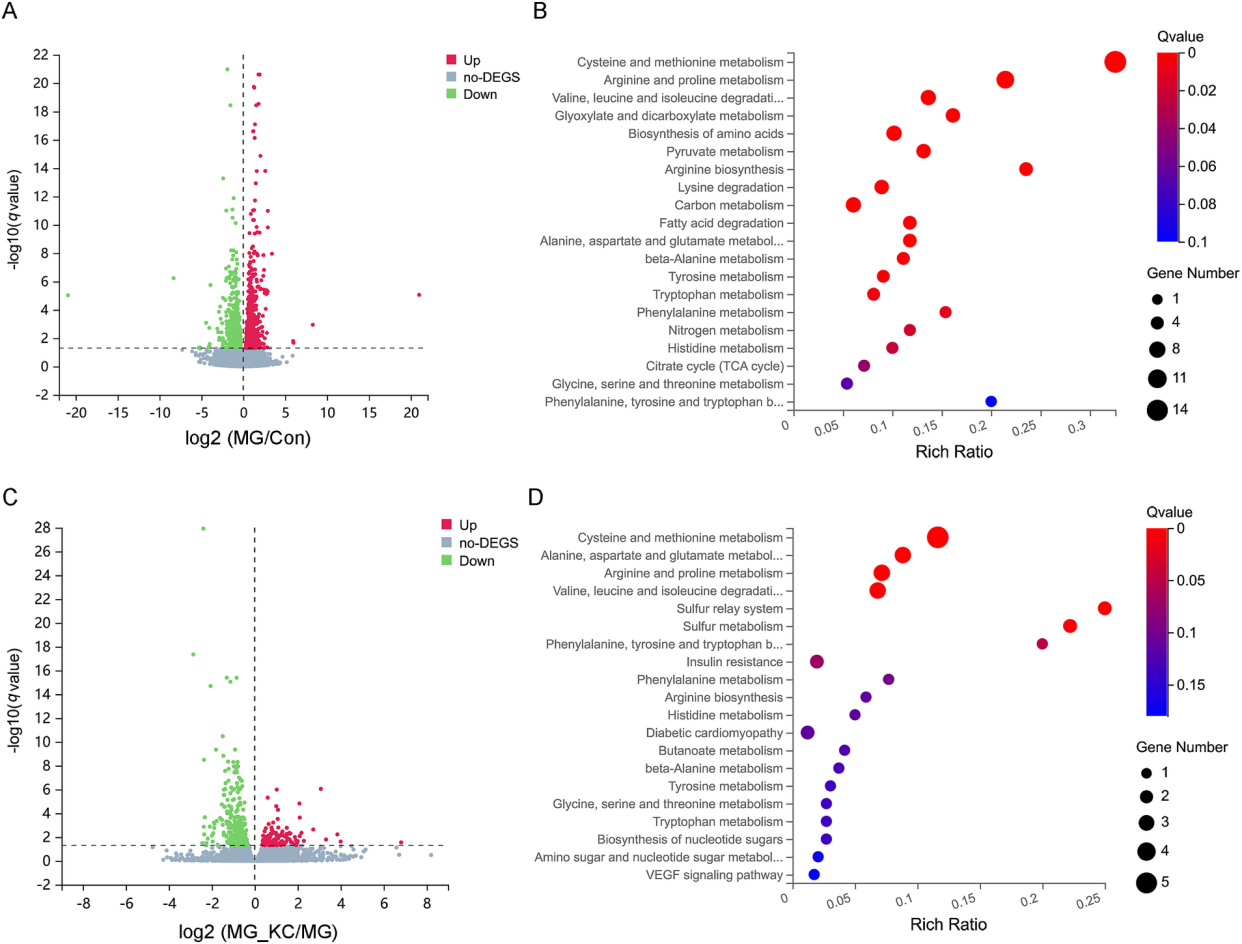
Effect of dietary *Bacillus subtilis* KC1 supplementation on the pulmonary transcriptional expression profile of MG-infected broilers. **(A)** Volcano plots showed the results of the pairwise comparisons of pulmonary genes in the Con and MG groups (*n* = 3). The significant genes are presented in red (up-regulated) or green (down-regulated). **(B)** The pathway enrichment analysis of significantly different genes in the Con and MG groups (*n* = 3). **(C)** Volcano plots showed the results of the pairwise comparisons of pulmonary genes in the MG and MG_KC groups (*n* = 3). The significant genes are presented in red (up-regulated) or green (down-regulated). **(D)** The pathway enrichment analysis of significantly different genes in the MG and MG_KC groups (*n* = 3). Con, control group; KC, *Bacillus subtilis* KC1 group; MG, *Mycoplasma gallisepticum* group; MG_KC, *Mycoplasma gallisepticum + Bacillus subtilis* KC1 group.

A total of 1,570 significantly differential genes in the lung were identified in the MG group compared to the Con group (Supplementary File S4). The volcano plots illustrated the global distribution of genes, revealing distinct differences in gene expression levels in the lung between the Con and MG groups (Figure 4A). KEGG pathway enrichment analysis indicated that 12 pathways related to amino acid metabolism were significantly enriched (Figure 4B). Furthermore, a total of 458 significantly differential genes in the lung were identified in the MG_KC group compared with the MG group (Supplementary File S5). The volcano plots illustrated the global distribution of genes, revealing distinct differences in gene expression levels in the lung between the MG and MG_KC groups (Figure 4C). KEGG pathway enrichment analysis indicated that five pathways related to amino acid metabolism were significantly enriched (Figure 4D). It is worth noting that four specific metabolic pathways, namely Cysteine and methionine metabolism, Arginine and proline metabolism, Valine, leucine and isoleucine degradation, as well as Alanine, aspartate and glutamate metabolism, are significantly enriched in both of the above enrichment analysis results (Figures 4B,D).

**Figure 5:**
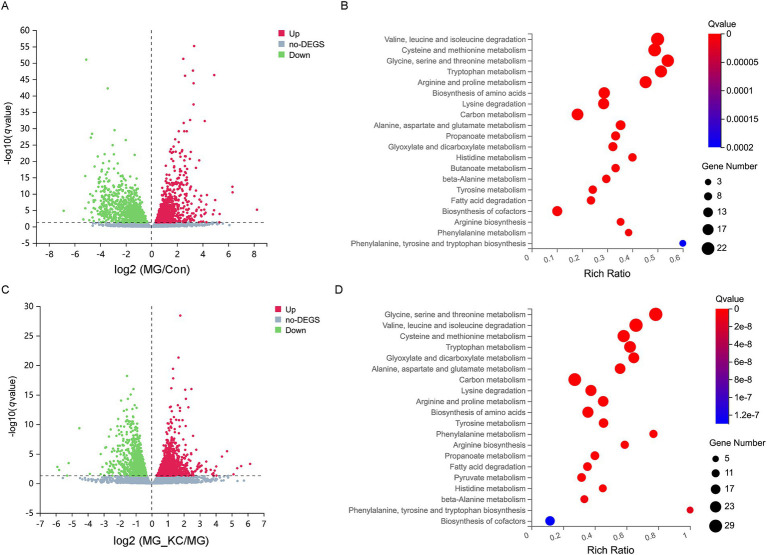
Effect of dietary *Bacillus subtilis* KC1 supplementation on the hepatic transcriptional expression profile of MG-infected broilers. **(A)** Volcano plots showed the results of the pairwise comparisons of hepatic genes in the Con and MG groups (*n* = 3). The significant genes presented in red (up-regulated) or green (down-regulated). **(B)** The pathway enrichment analysis of significantly different genes in the Con and MG groups (*n* = 3). **(C)** Volcano plots showed the results of the pairwise comparisons of hepatic genes in the MG and MG_KC groups (*n* = 3). The significant genes are presented in red (up-regulated) or green (down-regulated). **(D)** The pathway enrichment analysis of significantly different genes in the MG and MG_KC groups (*n* = 3). Con, control group; KC, *Bacillus subtilis* KC1 group; MG, *Mycoplasma gallisepticum* group; MG_KC, *Mycoplasma gallisepticum + Bacillus subtilis* KC1 group.

A total of 2,738 significantly differential genes in the liver were identified in the MG group compared with the Con group (Supplementary File S6). The volcano plots illustrated the global distribution of genes, revealing distinct differences in gene expression levels in the liver between the Con and MG groups (Figure 5A). KEGG pathway enrichment analysis indicated that 14 pathways related to amino acid metabolism were significantly enriched (Figure 5B). Furthermore, a total of 2,967 significantly differential genes in the liver were identified in the MG_KC group compared with the MG group (Supplementary File S7). The volcano plots illustrated the global distribution of genes, revealing distinct differences in gene expression levels in liver between the MG and MG_KC groups (Figure 5C). KEGG pathway enrichment analysis indicated that 14 pathways related to amino acid metabolism were significantly enriched (Figure 5D). It is worth noting that 14 metabolic pathways, including valine, leucine, and isoleucine degradation; cysteine and methionine metabolism; glycine, serine, and threonine metabolism; tryptophan metabolism; arginine and proline metabolism; biosynthesis of amino acids; lysine degradation; alanine, aspartate, and glutamate metabolism; histidine metabolism; beta-alanine metabolism; tyrosine metabolism; arginine biosynthesis; phenylalanine metabolism; and phenylalanine, tyrosine, and tryptophan biosynthesis, are significantly enriched in both of the above enrichment analysis results (Figures 5B,D).

**Figure 6:**
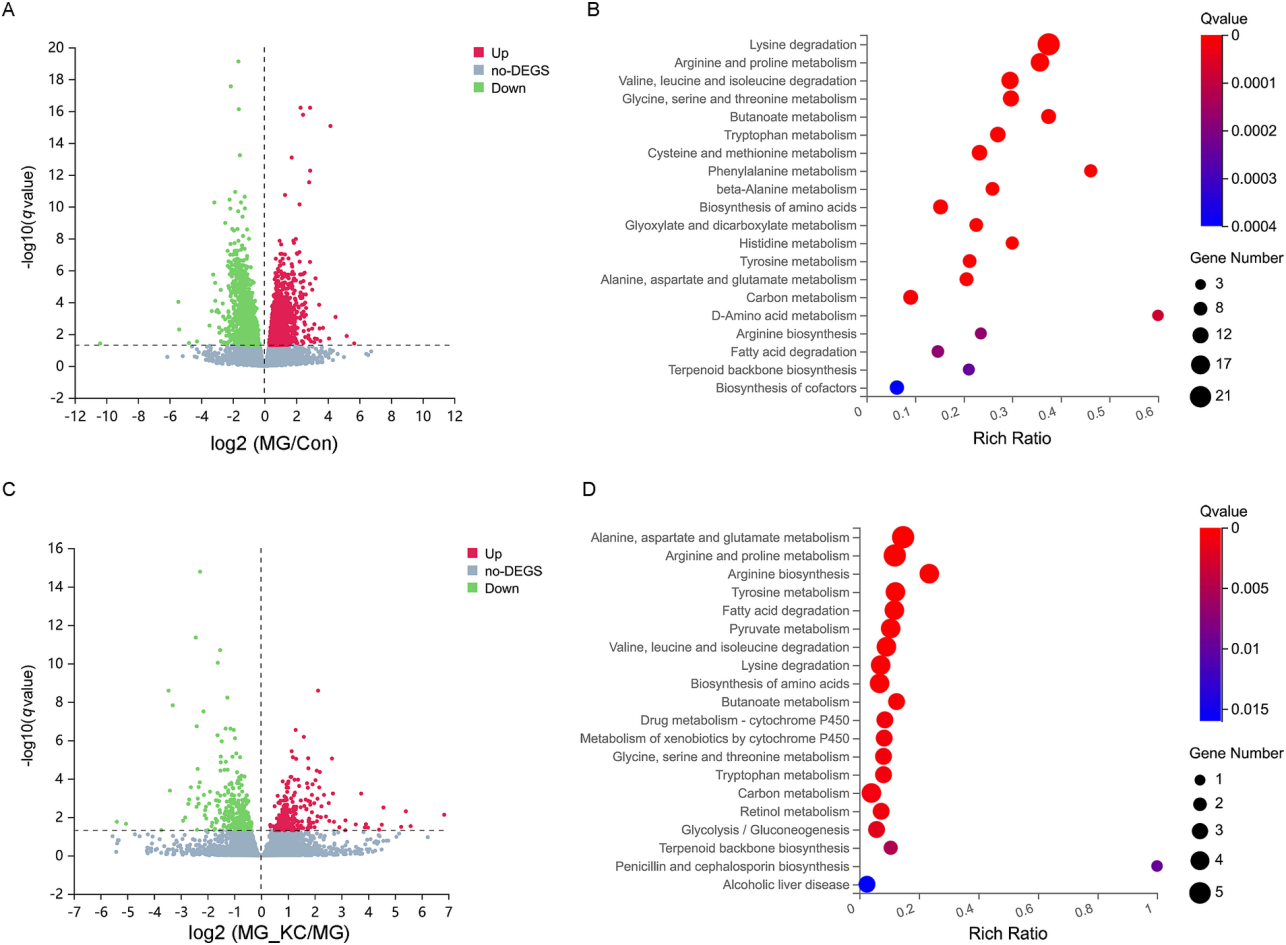
Effect of dietary *Bacillus subtilis* KC1 supplementation on the ileal transcriptional expression profile of MG-infected broilers. **(A)** Volcano plots showed the results of the pairwise comparisons of ileal genes in the Con and MG groups (*n* = 3). The significant genes are presented in red (up-regulated) or green (down-regulated). **(B)** The pathway enrichment analysis of significantly different genes in the Con and MG groups (*n* = 3). **(C)** Volcano plots showed the results of the pairwise comparisons of ileal genes in the MG and MG_KC groups (*n* = 3). The significant genes are presented in red (up-regulated) or green (down-regulated). **(D)** The pathway enrichment analysis of significantly different genes in the MG and MG_KC groups (*n* = 3). Con, control group; KC, *Bacillus subtilis* KC1 group; MG, *Mycoplasma gallisepticum* group; MG_KC, *Mycoplasma gallisepticum + Bacillus subtilis* KC1 group.

A total of 3,553 significantly differential genes in the ileum were identified in the MG group compared with the Con group (Supplementary File S8). The volcano plots illustrated the global distribution of genes, revealing distinct differences in gene expression levels in the ileum between the Con and MG groups (Figure 6A). KEGG pathway enrichment analysis indicated that 14 pathways related to amino acid metabolism were significantly enriched (Figure 6B). Furthermore, a total of 579 significantly differential genes in the ileum were identified in the MG_KC group compared with the MG group (Supplementary File S9). The volcano plots illustrated the global distribution of genes, revealing distinct differences in gene expression levels in ileum between the MG and MG_KC groups (Figure 6C). KEGG pathway enrichment analysis indicated that nine pathways related to amino acid metabolism were significantly enriched (Figure 6D). It is noteworthy that nine metabolic pathways, including lysine degradation, arginine and proline metabolism, valine, leucine, and isoleucine degradation, glycine, serine, and threonine metabolism, tryptophan metabolism, biosynthesis of amino acids, tyrosine metabolism, alanine, aspartate, and glutamate metabolism, and arginine biosynthesis, are significantly enriched in both of the aforementioned enrichment analysis results (Figures 6B,D). In summary, these results indicated that MG infection induced amino acids metabolic disturbance in chickens, while *B. subtilis* KC1 alleviated the amino acid metabolism disorder caused by MG.

## 4 Discussion

Some research indicates that *B. subtilis* as feed additives can enhance the growth performance of broilers (4, 5, 7). *B. subtilis* possesses the capability to secrete highly potent enzymes, including protease, lipase, and amylase, which efficiently break down complex carbohydrates, thereby enhancing nutrient digestibility and subsequently providing animals with an enriched nutritional supply (20). In addition, *B. subtilis* has been shown to have the ability to enhance disease resistance in animals. For example, dietary *B. subtilis* HW2 supplementation enhanced growth performance and alleviated gut injury in broilers under necrotic enteritis challenge (5). Dietary administration of *B. subtilis* PB6 improved growth performance and gut health in broilers challenged with *Clostridium perfringens* (21). Similar to previous studies, in the present study, *B. subtilis* KC1 significantly increased final body weight, increased ADG and decreased FCR in broilers. Furthermore, *B. subtilis* KC1 alleviated the negative effects of MG infection on growth performance. These results suggested that *B. subtilis* KC1 has the potential as a feed additive to resist MG infection.

The MG can not only cause respiratory tract damage in chickens, but also break through the respiratory barrier and cause damage to other organs through blood circulation (22–24). Oxidative stress and inflammatory damage are considered to be important mechanisms of host injury caused by MG infection (25, 26). *Bacillus subtilis* has been shown to possess a strong antioxidant capacity and to reduce inflammatory damage (27, 28). In the present study, MG infection induced systemic oxidative stress and a pro-inflammatory response, whereas *B. subtilis* KC1 alleviated these adverse effects caused by MG. These results suggested that *B. subtilis* KC1 may enhance the resistance of broilers to MG by regulating oxidative stress and inflammatory response.

An essential indicator for assessing the nutritional well-being of whole-body protein is the content of serum amino acids (29, 30). It has been reported that leucine, lysine and proline are vital in promoting poultry growth (30–33). In addition, arginine has been demonstrated to induce secretion of growth hormone and affect skeletal muscle development in chickens (34, 35). In the present study, metabolomics results revealed that MG infection significantly decreased the serum levels of leucine, lysine, proline, and arginine, whereas *B. subtilis* KC1 partially mitigated this amino acid imbalance induced by MG infection. This finding partly elucidates why MG infection leads to weight loss in broilers and how *B. subtilis* KC1 alleviates this weight loss in MG-infected broilers. It has been indicated that tryptophan and its metabolites play an important role in alleviating inflammatory damage and improving barrier function (36). In the present study, MG infection significantly reduced the content of tryptophan in serum, whereas *B. subtilis* KC1 restored the content of tryptophan. This indicates that tryptophan metabolism may play an important role in the process of MG infection, but its detailed mechanism remains to be further studied.

In order to explore the mechanism of MG-induced abnormal amino acid metabolism in the host, transcriptomics was further carried out. Previous studies have conclusively demonstrated that MG can not only elicit respiratory tract injury but also cause abnormal liver function (37). Furthermore, MG has the capability to disrupt the gut microbiota, potentially affecting normal gut function (38). As a result, this study has specifically chosen lung, liver, and intestinal tissues, which are known to be associated with MG infection, for in-depth transcriptomic analysis. The transcriptomic results revealed that MG infection can lead to abnormal expression of genes related to amino acid metabolism in lung, liver, and ileum tissues, while *B. subtilis* KC1 can partially alleviate these abnormal gene expressions. This also explains why MG can induce abnormal amino acid metabolism in the host, and how *B. subtilis* KC1 can mitigate the amino acid abnormalities caused by MG infection. In this study, the metabolome and transcriptome were utilized to elucidate the reasons why *B. subtilis* KC1 alleviates the adverse effects of MG on the growth performance of broilers from a global perspective. This approach represents the innovation of the study. However, a limitation of the study is that it did not conduct in-depth mechanism research. Furthermore, previous studies have shown that gut microbiota are closely related to the growth performance and health of chickens, and *B. subtilis* can enhance the growth performance and immunity of chickens by modulating the gut microbiota (39–42). Another limitation of this study is that it did not investigate the gut microbiota to explore the protective effect of *B. subtilis* KC1 against MG infection in broiler chickens.

In summary, dietary supplementation with *B. subtilis* KC1 improves the growth performance of broilers subjected to MG infection. The positive effects of *B. subtilis* KC1 are associated with the improved amino acid metabolism of broilers.

## Data Availability

The RNA sequencing data has been deposited in the NCBI GEO database, accessible using the accession number GSE273913. Other datasets analyzed during the current study are available from the corresponding author upon reasonable request.

## Footnotes

^1^https://www.kegg.jp

## Supplementary material

SUPPLEMENTARY DATA SHEET 1: Details of metabolites identified by metabolomics.

SUPPLEMENTARY DATA SHEET 2: Details of differential metabolites between the Con group and MG group.

SUPPLEMENTARY DATA SHEET 3: Details of differential metabolites between the MG group and MG_KC group.

SUPPLEMENTARY DATA SHEET 4: Details of differential genes in the lung between the Con group and MG group.

SUPPLEMENTARY DATA SHEET 5: Details of differential genes in the lung between the MG group and MG_KC group.

SUPPLEMENTARY DATA SHEET 6: Details of differential genes in the liver between the Con group and MG group.

SUPPLEMENTARY DATA SHEET 7: Details of differential genes in the liver between the MG group and MG_KC group.

SUPPLEMENTARY DATA SHEET 8: Details of differential genes in the ileum between the Con group and MG group.

SUPPLEMENTARY DATA SHEET 9: Details of differential genes in the ileum between the MG group and MG_KCgroup.

## References

[1] Guo Y Miao Y Chen H Wang K Wang S Wang R. Revealing the mechanism: the influence of Baicalin on M1/M2 and Th1/Th2 imbalances in *mycoplasma gallisepticum* infection. Poult Sci. (2024) 103:104145. doi: 10.1016/j.psj.2024.104145, PMID: 39127004 PMC11367134

[2] Wei X Zhong Q Wang D Yan Z Liang H Zhou Q. Epidemiological investigations and multilocus sequence typing of *Mycoplasma gallisepticum* collected in China. Poult Sci. (2023) 102:102930. doi: 10.1016/j.psj.2023.102930, PMID: 37716233 PMC10507435

[3] Wu Z Ding L Bao J Liu Y Zhang Q Wang J. Co-infection of *Mycoplasma gallisepticum* and *Escherichia coli* triggers inflammatory injury involving the IL-17 signaling pathway. Front Microbiol. (2019) 10:2615. doi: 10.3389/fmicb.2019.02615, PMID: 31803158 PMC6872679

[4] Gao Z Wu H Shi L Zhang X Sheng R Yin F. Study of *Bacillus subtilis* on growth performance, nutrition metabolism and intestinal microflora of 1 to 42 d broiler chickens. Anim Nutr. (2017) 3:109–13. doi: 10.1016/j.aninu.2017.02.002, PMID: 29767043 PMC5941101

[5] Chen P Lv H Du M Liu W Che C Zhao J. *Bacillus subtilis* HW2 enhances growth performance and alleviates gut injury via attenuation of endoplasmic reticulum stress and regulation of gut microbiota in broilers under necrotic enteritis challenge. Poult Sci. (2024) 103:103661. doi: 10.1016/j.psj.2024.103661 38547540 PMC11000119

[6] Ji L Zhang L Liu H Shen J Zhang Y Lu L. *Bacillus subtilis* M6 improves intestinal barrier, antioxidant capacity and gut microbial composition in AA broiler. Front Nutr. (2022) 9:965310. doi: 10.3389/fnut.2022.965310, PMID: 36061900 PMC9428444

[7] Nguyen A Nguyen D Tran M Nguyen L Nguyen A Phan T. Isolation and characterization of *Bacillus subtilis* CH16 strain from chicken gastrointestinal tracts for use as a feed supplement to promote weight gain in broilers. Lett Appl Microbiol. (2015) 60:580–8. doi: 10.1111/lam.12411, PMID: 25754534

[8] Menconi A Morgan M Pumford N Hargis B Tellez G. Physiological properties and *Salmonella* growth inhibition of probiotic *Bacillus* strains isolated from environmental and poultry sources. Int J Bacteriol. (2013) 60:958408:580–8. doi: 10.1155/2013/958408 PMC4745483 26904728

[9] Bilal M Achard C Barbe F Chevaux E Ronholm J Zhao X. *Bacillus pumilus* and *Bacillus subtilis* promote early maturation of cecal microbiota in broiler chickens. Microorganisms. (2021) 9:1899. doi: 10.3390/microorganisms9091899, PMID: 34576794 PMC8465073

[10] Neijat M Habtewold J Shirley R Welsher A Barton J Thiery P. *Bacillus subtilis* strain DSM 29784 modulates the cecal microbiome, concentration of short-chain fatty acids, and apparent retention of dietary components in shaver white chickens during grower, developer, and laying phases. Appl Environ Microbiol. (2019) 85:e00402–19. doi: 10.1128/AEM.00402-19, PMID: 31076425 PMC6606875

[11] Dong Y Li R Liu Y Ma L Zha J Qiao X. Benefit of dietary supplementation with *Bacillus subtilis* BYS2 on growth performance, immune response, and disease resistance of broilers. Probiotics Antimicrob Proteins. (2020) 12:1385–97. doi: 10.1007/s12602-020-09643-w, PMID: 32128666

[12] Lee J Kye J Park S Shim B Yoo S Hwang E. *Bacillus subtilis* spores as adjuvants against avian influenza H9N2 induce antigen-specific antibody and T cell responses in white Leghorn chickens. Vet Res. (2020) 51:68. doi: 10.1186/s13567-020-00788-8, PMID: 32448402 PMC7245620

[13] Cai H Luo S Zhou Q Yan Z Liu Q Kang Z. Effects of *Bacillus subtilis* and coccidiosis vaccine on growth indices and intestinal microbiota of broilers. Poult Sci. (2022) 101:102091. doi: 10.1016/j.psj.2022.102091, PMID: 36095864 PMC9472081

[14] Schrimpe-Rutledge A Codreanu S Sherrod S McLean J. Untargeted metabolomics strategies-challenges and emerging directions. J Am Soc Mass Spectrom. (2016) 27:1897–905. doi: 10.1007/s13361-016-1469-y, PMID: 27624161 PMC5110944

[15] Qiu S Cai Y Yao H Lin C Xie Y Tang S. Small molecule metabolites: discovery of biomarkers and therapeutic targets. Signal Transduct Target Ther. (2023) 8:132. doi: 10.1038/s41392-023-01399-3, PMID: 36941259 PMC10026263

[16] Wang J Ishfaq M Miao Y Liu Z Hao M Wang C. Dietary administration of *Bacillus subtilis* KC1 improves growth performance, immune response, heat stress tolerance, and disease resistance of broiler chickens. Poult Sci. (2022) 101:101693. doi: 10.1016/j.psj.2021.101693, PMID: 35066384 PMC8789536

[17] National Research Council (NRC). Nutrient requirements of poultry. 9th ed. Washington: National Academy Press (1994).

[18] Wen J Cai H Guo Y Qi G Chen J Zhang G. China NY/T 33-2004. Feeding standard of chicken. In: China NongYe Biaozhun/Tuijian-33-2004. Beijing: China Agricultural Publisher (2004)

[19] Love M Huber W Anders S. Moderated estimation of fold change and dispersion for RNA-seq data with DESeq2. Genome Biol. (2014) 15:550. doi: 10.1186/s13059-014-0550-8, PMID: 25516281 PMC4302049

[20] Khochamit N Siripornadulsil S Sukon P Siripornadulsil W. Antibacterial activity and genotypic-phenotypic characteristics of bacteriocin-producing *Bacillus subtilis* KKU213: potential as a probiotic strain. Microbiol Res. (2015) 170:36–50. doi: 10.1016/j.micres.2014.09.004, PMID: 25440998

[21] Liu Y Zhang S Luo Z Liu D. Supplemental *Bacillus subtilis* PB6 improves growth performance and gut health in broilers challenged with *Clostridium perfringens*. J Immunol Res. (2021) 2021:2549541. doi: 10.1155/2021/2549541, PMID: 34746321 PMC8566084

[22] Wang S Guo L Gu F Bao J Guo Y Zhang Y. Quercetin restores respiratory mucosal barrier dysfunction in *Mycoplasma gallisepticum*-infected chicks by enhancing Th2 immune response. Phytomedicine. (2024) 133:155953. doi: 10.1016/j.phymed.2024.155953, PMID: 39154527

[23] Wang K Miao Y Liu W Ishfaq M Bao J Jin X. *Lactobacillus salivarius* ameliorates *Mycoplasma gallisepticum*-induced inflammation via the JAK/STAT signaling pathway involving respiratory microbiota and metabolites. Poult Sci. (2024) 103:103942. doi: 10.1016/j.psj.2024.103942, PMID: 38908119 PMC11246048

[24] Ishfaq M Zhang W Liu Y Wang J Wu Z Shah S. Baicalin attenuated *Mycoplasma gallisepticum*-induced immune impairment in chicken bursa of fabricius through modulation of autophagy and inhibited inflammation and apoptosis. J Sci Food Agric. (2021) 101:880–90. doi: 10.1002/jsfa.10695, PMID: 32729138

[25] Ishfaq M Wu Z Wang J Li R Chen C Li J. Baicalin alleviates *Mycoplasma gallisepticum*-induced oxidative stress and inflammation via modulating NLRP3 inflammasome-autophagy pathway. Int Immunopharmacol. (2021) 101:108250. doi: 10.1016/j.intimp.2021.108250 34656906

[26] Wang S Jin X Chen H Han M Bao J Niu D. Quercetin alleviates *Mycoplasma gallisepticum*-induced inflammatory damage and oxidative stress through inhibition of TLR2/MyD88/NF-κB pathway in vivo and in vitro. Microb Pathog. (2023) 176:106006. doi: 10.1016/j.micpath.2023.106006, PMID: 36746315

[27] Zou X Zhang M Tu W Zhang Q Jin M Fang R. *Bacillus subtilis* inhibits intestinal inflammation and oxidative stress by regulating gut flora and related metabolites in laying hens. Animal. (2022) 16:100474. doi: 10.1016/j.animal.2022.100474 35220172

[28] Bai W Zhang F He T Su P Ying X Zhang L. Dietary probiotic *Bacillus subtilis* strain fmbj increases antioxidant capacity and oxidative stability of chicken breast meat during storage. PLoS One. (2016) 11:E0167339. doi: 10.1371/journal.pone.0167339, PMID: 27907152 PMC5132206

[29] Liao S Regmi N Wu G. Homeostatic regulation of plasma amino acid concentrations. Front Biosci. (2018) 23:640–55. doi: 10.2741/4610, PMID: 28930566

[30] Liang Y Zheng X Wang J Yang H Wang Z. Different amino acid supplementation patterns in low-protein diets on growth performance and nitrogen metabolism of goslings from 1 to 28 days of age. Poult Sci. (2023) 102:102395. doi: 10.1016/j.psj.2022.102395, PMID: 36571878 PMC9803941

[31] Xie W Fu Z Pan N Yan H Wang X Gao C. Leucine promotes the growth of squabs by increasing crop milk protein synthesis through the TOR signaling pathway in the domestic pigeon (*Columba livia*). Poult Sci. (2019) 98:5514–24. doi: 10.3382/ps/pez296 31172174

[32] Jespersen J Richert S Cesar de P Oelschlager M Dilger R. Effects of lysine biomass supplementation on growth performance and clinical indicators in broiler chickens. Poult Sci. (2021) 100:100971. doi: 10.1016/j.psj.2020.12.068, PMID: 33516469 PMC7936182

[33] Lee C Song A Loh T Abdul R. Effects of lysine and methionine in a low crude protein diet on the growth performance and gene expression of immunity genes in broilers. Poult Sci. (2020) 99:2916–25. doi: 10.1016/j.psj.2020.03.013, PMID: 32475425 PMC7597739

[34] Brugaletta G Zampiga M Laghi L Indio V Oliveri C De Cesare A. Feeding broiler chickens with arginine above recommended levels: effects on growth performance, metabolism, and intestinal microbiota. J Anim Sci Biotechnol. (2023) 14:33. doi: 10.1186/s40104-023-00839-y 36864475 PMC9983211

[35] Wang R Li K Sun L Jiao H Zhou Y Li H. L-arginine/nitric oxide regulates skeletal muscle development via muscle fbre-specifc nitric oxide/mTOR pathway in chickens. Anim Nutr J. (2022) 10:68–85. doi: 10.1016/j.aninu.2022.04.010, PMID: 35647326 PMC9125674

[36] Zhao C Hu X Bao L Wu K Feng L Qiu M. Aryl hydrocarbon receptor activation by *Lactobacillus reuteri* tryptophan metabolism alleviates *Escherichia coli*-induced mastitis in mice. PLoS Pathog. (2021) 17:e1009774. doi: 10.1371/journal.ppat.1009774 34297785 PMC8336809

[37] Luo R Fan C Jiang G Hu F Wang L Guo Q. Andrographolide restored production performances and serum biochemical indexes and attenuated organs damage in *Mycoplasma gallisepticum*-infected broilers. Br Poult Sci. (2023) 64:164–75. doi: 10.1080/00071668.2022.2128987 36222587

[38] Wang J Ishfaq M Li J. *Lactobacillus salivarius* ameliorated *Mycoplasma gallisepticum*-induced inflammatory injury and secondary *Escherichia coli* infection in chickens: involvement of intestinal microbiota. Vet Immunol Immunopathol. (2021) 233:110192. doi: 10.1016/j.vetimm.2021.110192, PMID: 33476924

[39] Chen S Luo S Yan C. Gut microbiota implications for health and welfare in farm animals: a review. Animals. (2021) 12:93. doi: 10.3390/ani12010093, PMID: 35011199 PMC8749645

[40] Yan C Xiao J Li Z Liu H Zhao X Liu J. Exogenous fecal microbial transplantation alters fearfulness, intestinal morphology, and gut microbiota in broilers. Front Vet Sci. (2021) 8:706987. doi: 10.3389/fvets.2021.706987, PMID: 34660756 PMC8517117

[41] Xu Y Yu Y Shen Y Li Q Lan J Wu Y. Effects of *Bacillus subtilis* and *Bacillus licheniformis* on growth performance, immunity, short chain fatty acid production, antioxidant capacity, and cecal microflora in broilers. Poult Sci. (2021) 100:101358. doi: 10.1016/j.psj.2021.101358, PMID: 34358955 PMC8350532

[42] Qiu K Li C Wang J Qi G Gao J Zhang H. Effects of dietary supplementation with *Bacillus subtilis*, as an alternative to antibiotics, on growth performance, serum immunity, and intestinal health in broiler chickens. Front Nutr. (2021) 8:786878. doi: 10.3389/fnut.2021.786878, PMID: 34917643 PMC8668418

